# Reduction-Driven Modulation of Relaxivity in Graphene Oxide Derivatives

**DOI:** 10.1021/acsomega.6c04502

**Published:** 2026-07-31

**Authors:** Giulia Fioravanti, Laura Torrieri Di Tullio, Salvatore Mamone, Paola Fattibene, Marcello Alecci, Silvia Colacicchi, Angelo Galante

**Affiliations:** † Department of Physical and Chemical Sciences, 9303 University of L’Aquila, 67100 L’Aquila, Italy; ‡ Istituto Superiore di Sanità, Viale Regina Elena 299, 00161 Rome, Italy; § Department of Life, Health and Environmental Sciences, University of L’Aquila, 67100 L’Aquila, Italy; ∥ Gran Sasso National Laboratory (LNGS), National Institute for Nuclear Physics (INFN), 67100 L’Aquila, Italy; ⊥ CNR-SPIN, c/o Department of Physical and Chemical Sciences, 67100 L’Aquila, Italy

## Abstract

Graphene oxide (GO)-based nanomaterials have emerged as promising platforms in biomedicine, particularly for their potential use as carriers of magnetic resonance imaging (MRI) contrast agents (CAs). In this study, we investigate the MRI CA performance of reduced graphene oxide and its dependence on the degree of reduction. To ensure full control, GO was synthesized in-house via a modified Hummers’ method and subsequently reduced by two distinct approaches: mild thermal reduction (rGO1) and selective chemical reduction with sodium borohydride (rGO2). Comprehensive physicochemical characterization, including Fourier-transform infrared (FTIR) spectroscopy, Raman spectroscopy, X-ray photoelectron spectroscopy, differential scanning calorimetry, and thermogravimetric analysis (TGA), was performed to evaluate the structural changes induced by each reduction process. The MRI performance of GO, rGO1, and rGO2 was assessed at 1.0 T alongside electron paramagnetic resonance (EPR) measurements, while inductively coupled plasma-mass spectrometry (ICP-MS) quantified residual manganese and iron impurities. EPR and ICP-MS demonstrated that these impurities made a negligible contribution to the relaxivity. Our findings reveal a significant enhancement in MRI relaxivities with an increasing degree of reduction. Chemically reduced rGO2 exhibits the highest longitudinal relaxivity (*r*_1_), approximately 3.9-fold greater than that of pristine GO, and transverse relaxivity (*r*_2_), approximately 4.6-fold greater. Strong linear correlations between relaxivities and XPS-derived structural parameters, including the C/O ratio and C–C/C=C content, indicate that carbon structural defects govern MRI contrast performance. Overall, these results establish a framework for the rational design of rGO-based CAs through controlled reduction of two-dimensional carbon materials, enabling optimized imaging performance and supporting future biomedical translation.

## Graphical abstract

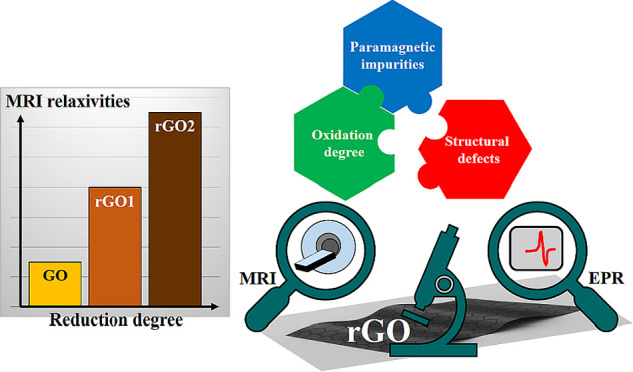

## 1 Introduction

Among the most intriguing 2D materials,^1^ a special mention goes to graphene oxide (GO), a highly oxidized form of chemically modified graphene that consists of a single layer of carbon atoms, with the lateral size of the GO flakes ranging from the nano- to microscale, decorated with oxygenated groups (carboxylic acids, epoxides, and hydroxyl), with the Lerf–Klinowski structural model the most currently accepted in the literature.^2^ Reduced graphene oxide (rGO) can be obtained by the physical or chemical reduction of GO. It may retain some oxygenated groups in its graphene skeleton, allowing it to exhibit a variable hydrophobicity depending on the reduction degree.^3^

GO and rGO are attractive nanomaterials for biomedical applications owing to their high aqueous dispersibility, scalable production, tunable surface chemistry, and ease of functionalization. These characteristics have enabled their use in a broad range of applications including drug delivery,^4^ biosensing, tissue engineering,^5–7^ antimicrobial coatings,^8,9^ antioxidant and anti-inflammatory therapies,^10–13^ as well as photocatalytic systems.^14–16^ Recent studies have further demonstrated that the biological performance of GO-based materials strongly depends on the degree of oxidation, defect density, and surface functionalization, emphasizing the need for accurate physicochemical characterization before translating these materials into biomedical applications.^17^

Magnetic resonance imaging (MRI) is a noninvasive technique offering submillimeter spatial resolution and excellent contrast based on water content and longitudinal (T_1_) and transverse (T_2_) relaxation times. However, their sensitivity is relatively lower compared to other imaging methods. MRI contrast agents (CAs) have been actively explored to enhance T_1_ and T_2_ molar relaxivities, thus improving contrast,^18^ with gadolinium-based CAs playing the main role in preclinical and clinical settings.^19^

Unlike conventional gadolinium-based CAs, graphene-derived materials offer the possibility of modulating relaxometric properties through controlled variations of their chemical composition and defect structure, potentially reducing the need for externally introduced paramagnetic ions. This has stimulated increasing interest in understanding the intrinsic origin of their MRI contrast mechanisms and identifying the physicochemical parameters governing water proton relaxation.^20,21^ Their paramagnetic behavior arises from three main features: (i) carbon structural defects located on the edges and holes (dangling bonds); (ii) oxygenated functional groups; and (iii) residual metallic impurities anchored to the graphene skeleton due to the specific synthetic pathway. Residual metallic contaminants originating from graphite precursors or oxidation/reduction procedures may substantially alter the measured magnetic response, thereby masking the intrinsic contribution of the graphene lattice. Consequently, distinguishing the respective roles of structural defects, oxygen-containing functional groups, and trace paramagnetic metals remains one of the major challenges in interpreting the relaxometric behavior of GO-derived materials.

Conflicting interpretations exist in the literature: one study attributed GO paramagnetism primarily to dangling-bond carbon defects,^22^ while another highlighted the combined role of oxidation degree and intercalated Mn­(II).^23^ A recent critical review has also underlined the importance of rigorous characterization to avoid misinterpretation of GO properties.^17^ In our previous work, we disentangled these contributions in highly purified GO, showing that residual contaminants do not govern intrinsic relaxivity when proper cleaning is applied.^24^

In this work, we aimed to advance our understanding of the interplay among the above three features in the paramagnetism of GO-based materials. To this purpose, for the first time, the metal impurities were reduced to avoid any significant effect on relaxivities,^24^ and we modulated the degree of oxidation showing a correlation between the residual functional groups on rGO and the MRI relaxivities.

The pristine GO was reduced to rGO through either a thermal treatment (batch rGO1) or a chemical treatment (batch rGO2), as described below. Figure 1 presents a schematic illustration of the reduction process from GO (brown aqueous dispersion) to rGO (black aqueous dispersion).

**Figure 1:**
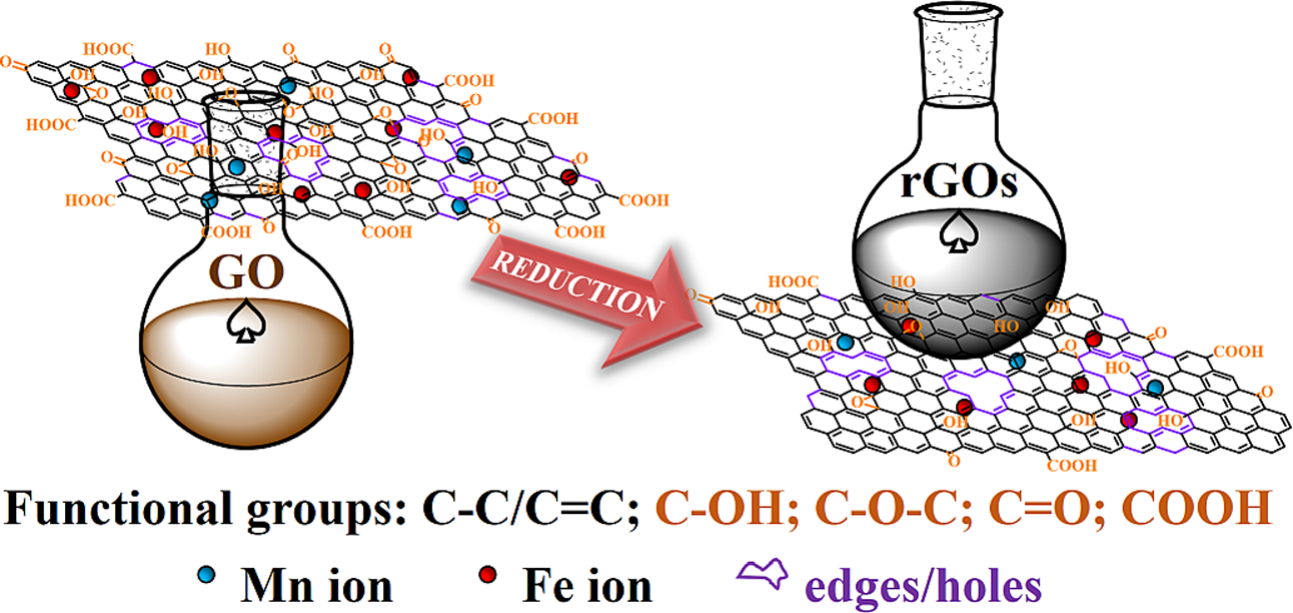
Schematic illustration of the reduction process from GO to rGOs.

The scheme highlights that the reduced materials still retain residual oxygen-containing functional groups (hydroxyl, epoxy, and carboxyl), structural defects (e.g., holes and dangling bonds), and paramagnetic metal contaminants.

In the GO, rGO1, and rGO2 batches, the degree of reduction, the presence of paramagnetic impurities (Mn and Fe), and the quantification of the residual surface functionalities were determined using a comprehensive range of physicochemical and spectrometry/spectroscopy techniques.

Our extensive analysis and findings reveal an increased MRI relaxivity upon reduction, particularly in the chemically reduced rGO2 sample, with an observed highest longitudinal (about 3.9 times) and transverse (about 4.6 times) relaxivity with respect to that of the pristine GO sample.

## 2 Materials and Methods

GO was synthesized using a modified Hummers method,^25^ as reported in the Supporting Information file. The first rGO1 batch was prepared by a mild thermal reduction, carried out on a dry sample kept in a common laboratory oven (80 °C for a total of 24 h).^26^ The second rGO2 batch was obtained by chemical reduction with sodium borohydride treatment following the protocol reported in ref 27.

The chemical–physical properties of the GO, rGO1, and rGO2 samples were fully characterized by Fourier-transform infrared (FTIR) spectroscopy, Raman spectroscopy (RS), differential scanning calorimetry (DSC), and thermogravimetric analysis (TGA). The experimental conditions and results are reported in the Supporting Information. X-ray photoelectron spectroscopy (XPS) was employed to quantify the residual surface functionalities after the thermal and chemical reduction processes. Manganese and iron contamination were determined by inductively coupled plasma-mass spectrometry (ICP-MS). Room- and cryogenic-temperature X-band electron paramagnetic resonance (EPR) spectroscopy and room-temperature 1.0 T MRI studies were carried out to investigate and quantify the paramagnetic species (Mn, Fe) and the water relaxivities of the GO, rGO1, and rGO2 materials, respectively.

### 2.1 X-ray Photoelectron Spectroscopy

XPS spectra were acquired under ultrahigh vacuum (UHV) conditions using a PHI ESCA system equipped with an Mg Kα X-ray source (*h*ν = 1253.6 eV) and a hemispherical electron energy analyzer. The peak areas were corrected using the appropriate Relative Sensitivity Factors prior to the determination of the atomic concentrations. The XPS spectra were analyzed by fitting the components with Voigt line shapes and Shirley-type backgrounds.^28^ The samples were deposited on a Si substrate (1 cm^2^, SiO_2_, oxide thickness 270 nm). The substrate was previously sonicated 10 min with acetone (Sigma-Aldrich product N. 24201, >99%), 10 min with isopropyl alcohol (Sigma-Aldrich product N. W292907, 99.7%), and 10 min with diluted basis Piranha (1:3:4 of H_2_O_2_/NH_4_OH/H_2_O) (Hydrogen peroxide, Sigma-Aldrich product N. 95294, 30%; Ammonia solution, Sigma-Aldrich product N. 05002, 33%) and then finally rinsed with Milli-Q water. The GO/rGO samples were deposited by drop casting a dilute aqueous solution (1.0 mg/mL, volume of 20 μL) of the material on the substrate.

### 2.2 Inductively Coupled Plasma-Mass Spectrometry (ICP-MS)

Manganese and iron contamination were determined by the Thermo Fisher iCAP TQe ICP-MS instrument. For ICP-MS analysis, 8 mg of samples was mineralized with 2.65 mL of ultrapure aqua regia (nitric and hydrochloric acid, 1:3; Merck Life Science S.r.l., Italy) at 180 °C for 20 min by means of a microwave digestor (Ethos One, Milestone, Bergamo, Italy) in closed polytetrafluoroethylene (PTFE) reactors. For aqua regia: Suprapur HCl 30% (Merck Life Science S.r.l., product N. 1.00318); Suprapur HNO_3_ 65% (Merck Life Science S.r.l., product N. 1.00441). The microwave protocol consists of a heating ramp 0–180 °C in 10 min, thermostating at 180° for 20 min, and cooling down at room temperature (RT). Mineralized samples were recovered with Milli-Q water, diluted to 100 mL, filtered through a 0.2 μm recycled cellulose syringe filter, and subsequently diluted by a factor of 2. Measurements were performed in triplicate experiments. Mono- and multielement stock TraceCERT solutions for ICP calibration were purchased from Sigma-Aldrich (Mn standard, product N. 74128; Fe standard product N. 43149). Matrix effect and instrumentation drift were monitored using a 20 μg/L Rhodium solution as the Internal Standard for all the analyzed solutions.^29^ All the standard solutions were prepared in 1% of aqua regia.

### 2.3 Electron Paramagnetic Resonance

Continuous Wave EPR (CW-EPR) measurements were performed at RT and at cryogenic temperature (100 K), with a Bruker X-band (9.81 GHz) spectrometer (Bruker Biospin S.r.l., Milano, Italy) equipped with a Bruker 3122SHQE cavity resonator. The temperature of 100 K was achieved and stabilized by a nitrogen Bruker VT system to enhance the detection of the Fe signal. A quartz EPR dewar was inserted in the cavity resonator to allow nitrogen flux. Spectrum acquisition parameters at RT were set as follows: central magnetic field 350 mT; data points 2048; modulation frequency 100 kHz; and scan range 20 mT or 140 mT. Modulation amplitudes of 0.1 mT or 1 mT and microwave powers of 15 mW or 1 mW were employed, depending on the required spectral resolution and signal amplification. For the 100 K EPR measurements, to better visualize the broader signals and to maintain stability during the measurements, the scan range was 330 mT, the modulation amplitude was 0.8 mT, and the power was 1 mW. Quartz Suprasil EPR tubes of 3 mm internal diameter (ATS Life Sciences Wilmad, NJ, USA, Suprasil EPR tubes, ID3) were used for all measurements. Solid-state GO/rGO1/rGO2 samples of known mass were directly inserted into the EPR tubes for measurements. Simulated RT GO/rGO1/rGO2 EPR spectra were generated by the MATLAB toolbox Easyspin v. 6.0.0 – dev.51 ^30^ using the available “pepper” function to reproduce the experimental data. We estimated the number of centers for the carbon and Mn­(II) defects using the double integration (DI) of the simulated line, normalized to the sample mass and spin multiplicity S­(S+1), where S is the electron spin. The spin multiplicity is three-fourths for the carbon defect and 35/4 for the Mn­(II) whose electron spins are half and 5/2, respectively.^31^ The 100 K EPR spectra were used to assess Fe content. Due to its asymmetric line, we used an approximate method to estimate the quantity present by directly calculating the area below the experimental spectrum using the common rule of thumb area = *h*_pp_ • *lw*,^2^ where *lw* is the line width and *h*_pp_ is the peak-to-peak height.^24^ The results were corrected for the sample mass. For the broad signals, such as those from Mn­(II) and Fe­(III) centers, we estimate an error of approximately 50% in the spin number, due to spectral distortions. This approach is based on considerations widely supported in the literature.^31^ The error for the narrow Lorentzian single-line component was estimated to be about 10%.

### 2.4 Magnetic Resonance Imaging

The GO, rGO1, and rGO2 samples suitable for MRI were prepared in an aqueous solution (Milli-Q water) at concentrations ranging from 0.01 to 1 mg/mL, using 2 mL PTFE/silicone septa glass vials (diameter 11.6 mm and height 31.4 mm) by sonicating up to 30 min before measurements (with Ultrasound Bath LBS 2; frequency: 59 kHz; and peak power: 450 W). MRI was performed with a 1.0 T preclinical M3 Aspect Imaging scanner (proton frequency of 45 MHz). The solenoid radiofrequency coil (3.5 cm inner diameter and 8 cm length) allowed the insertion of up to four vials at the same time. The T_1_ relaxation time was measured from axial Spin Echo images with a Field of View of 32 × 32 mm^2^; 1 mm in-plane resolution; 1 mm slice thickness; TE = 4.1 ms; and 18 different TR values (TR = 100, 150, 200, 250, 300, 350, 400, 450, 500, 750, 1000, 1500, 2000, 2500, 3000, 3500, 4000, and 5000 ms; two more values were used for the MnCl_2_ samples: TR = 30 and 50 ms). T_1_ maps were calculated by the exponential signal recovery within each image voxel, and the T_1_ value of each sample was given by the average of the corresponding vial’s voxels. The unavailability of multiecho Spin Echo MRI sequences prevented the acquisition of T_2_ relaxation mapping from images. Thus, we used a whole-sample Fast Spin Echo pulse sequence with echo train length equal to 128 and the gradients disabled, allowing a CPMG (Carr-Purcell-Meiboom-Gill) acquisition of each single vial. In our experimental conditions, multiple echoes from a single vial (TE = 4.4 ms) were acquired, finally extracting T_2_ from the echoes’ amplitude exponential decay. Voxel intensities and CPMG echoes fits were performed by means of in-house Matlab scripts (The MathWorks, Inc., Natick, MA, USA) using a 3-parameter monoexponential fitting based on the Levenberg–Marquardt nonlinear least-squares’ algorithm. All MRI measurements were done at 22 ± 1 °C. The relaxivities of the samples are expressed as a function of the concentration (mg/mL) due to the nonstoichiometric nature of the materials. The molar relaxivities *r*_1_ and *r*_2_ (mg/mL·s)^−1^ were calculated from the slope of the linear regression of 1/T_1_ (s^–1^) and 1/T_2_ (s^–1^) as a function of CA concentrations (mg/mL). MRI measurements were performed on three independently prepared dispersions.

## 3 Results and Discussion

Since the starting GO used in this study is not a commercial product, we report its chemical–physical characterization, together with those of the rGO1 and rGO2 samples.

The FTIR spectra of the samples were obtained in the 4000–400 cm^–1^ wavenumber range for the identification of functional groups and are reported in Figure S1 (Supporting Information). The GO spectrum confirmed the presence of hydroxyl and epoxy groups, as well as ether functionalities. These results are consistent with the existing literature.^32,33^ Thermally reduced rGO1 showed an enhanced C=C stretching signal while largely preserving oxygen-containing groups such as hydroxyls and epoxies:^34^ this mild reduction preserves most oxygen-containing functional groups on the graphene framework. In contrast, the chemically reduced rGO2 sample shows complete removal of C=O species, while the C=C signal is enhanced. The FTIR data clearly demonstrate progressive reduction from GO to rGO1 and rGO2: while rGO1 shows only partial removal of oxygenated groups, rGO2 exhibits a more efficient reduction, especially of carbonyl functionalities, while retaining some hydroxyl and epoxy groups. These trends are consistent with the expected behavior of thermal versus chemical reduction processes.

Raman spectra of GO, rGO1, and rGO2 are reported in Figure S2. The D/G band intensity ratio (I_D_/I_G_), a key indicator of graphitic defect density, was found to be 1.08 for GO, 1.04 for rGO1, and 0.91 for rGO2, respectively.^35^

DSC was performed to determine the thermal stability of the three samples, heating the samples from RT up to 350 °C (10 °C/min) in a nitrogen atmosphere, and the experimental results are reported in Figure S3. The intensity of a strong exothermic peak (at 198 °C), linked to the decomposition of stable oxygenated groups, decreases progressively from GO to rGO1 and rGO2.^36^

TGA records the change in a sample’s mass as the temperature increases and is useful for quantifying the efficiency of the reduction process. TGA results of graphite, GO, rGO1, and rGO2 are reported in Figure S4. The inclusion of graphite confirmed that the GO sample was well-oxidized, with no detectable residual graphitic content, confirming the efficiency of the initial oxidation process. rGO2 showed superior thermal stability and reduced oxygen content compared to the other samples.

The XPS survey spectra (Figure S5) confirmed that carbon and oxygen are the main elements present in all samples. The total surface elemental composition, including C 1s, O 1s, N 1s, S 2p, and B 1s contributions and the C/O ratio,^17^ are reported in Table 1. Very low amounts of N and S were detected in GO (approximately 1%), likely originating from the Hummers synthesis route, while 5.6% of B was observed in rGO2 due to the use of NaBH_4_ during the chemical reduction. Paramagnetic Mn and Fe impurities were below the sensitivity threshold (≃1%) of our detector.

**Table 1:** Surface Elemental Composition (%) Determined by XPS, Including C 1s, O 1s, N 1s, S 2p, and B 1s Contributions, together with the Calculated C/O Ratio for the GO, rGO1, and rGO2 Samples

	C 1s (%)	O 1s (%)	N 1s (%)	S 2p (%)	B 1s (%)	ratio C/O
**GO**	59.5	38.0	1.1	1.4	-	1.6
**rGO1**	70.5	29.5	-	-	-	2.4
**rGO2**	71.6	22.8	-	-	5.6	3.1

The estimated C/O ratio was 1.6 for GO, showing a high degree of oxidation of the material (this ratio varies according to the synthetic procedure followed and the oxidizing system chosen). For the reduced samples, the C/O ratio increased to higher values (almost twice for rGO2), as expected for reduced samples, where some oxygenated groups are cleaved. The XPS C 1s core level spectra for all of the samples are displayed in Figure 2. The spectra were fitted by the sum of five components assigned to C atoms belonging to (i) aromatic rings and hydrogenated carbon (C=C/C–C 284.8 eV), (ii) hydroxyl/epoxy groups (C–O 285.9 eV; C–O–C 286.9 eV), and (iii) carbonyl/carboxyl groups (C=O 288.2 eV; COOH 289.3 eV).^37^

**Figure 2:**
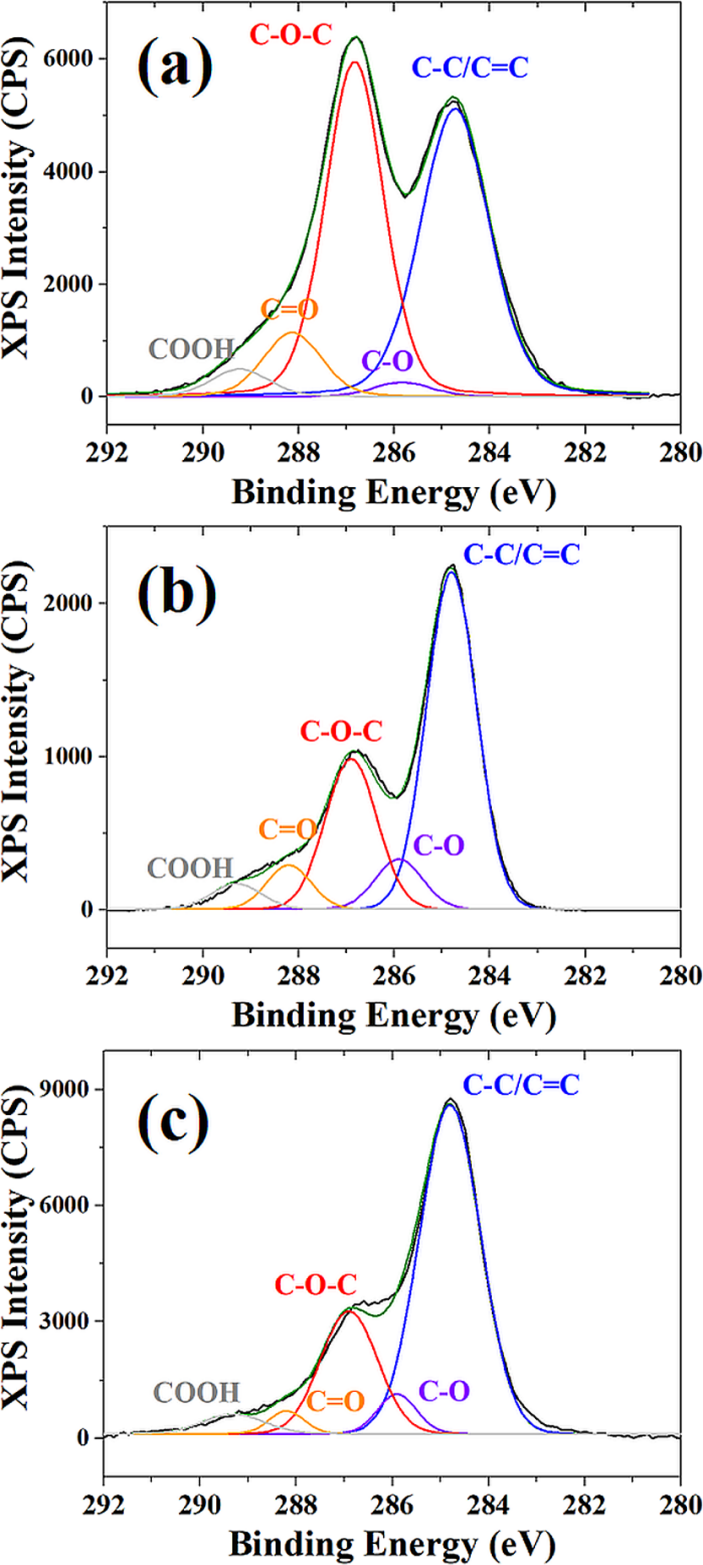
XPS C 1s spectra of (a) GO, (b) rGO1, and (c) rGO2 samples. The black lines represent the experimental spectra, and the colored ones the deconvoluted spectra to highlight the presence of five components (C–C/C=C; C–O; C–O–C; and C=O; COOH).

The XPS relative percentages of the deconvoluted C 1s peaks for the GO and rGO samples are reported in Table 2. In the GO sample (Figure 2a), the relative area percentages for C–C/C=C, C–O, C–O–C, C=O, and COOH were, respectively, 48.1%, 1.3%, 34.8%, 15.4%, and 0.4% (as reported in Table 2), which confirms the presence of a high number of oxygenated groups in the GO sample. Going into detail, we can see the contributions of the epoxy groups on the carbonaceous skeleton, which make the peak relative to the C–O–C very intense.

**Table 2:** Analysis of the Deconvoluted C 1s Peaks in Figure 2 and Relative Area Percentages

XPS C 1s fitting relative area percentage (binding energy, eV)					
samples	C–C/C=C (284.8 eV)	C–O (285.9 eV)	C–O–C (286.9 eV)	C=O (288.2 eV)	COOH (289.3 eV)
**GO**	48.1	1.3	34.8	15.4	0.4
**rGO1**	54.8	9.3	24.3	7.8	3.8
**rGO2**	65.9	5.0	22.7	2.5	3.9

From the analysis of the deconvoluted peaks, we observed an increase in the C–C/C=C contribution from 48.1% in GO to 54.8% in rGO1 and 65.9% in rGO2, confirming the partial reduction of GO to graphene-like sheets by removing oxygen-containing groups and recovering the conjugated structure. Compared to GO, the C–O single bond peak increased almost twice for rGO1 than for rGO2. This is likely due to the partial opening of epoxy rings, which converted to hydroxyl groups. The C–O–C epoxy signal became broader and decreased in intensity, dropping from 34.8% in the oxidized product to 22.7% in the chemically reduced rGO2 sample.

Both reduced samples, obtained by different synthesis methods, retained a significant percentage of epoxy groups, as corroborated by FTIR analysis, despite a pronounced reduction compared with GO. In the thermally reduced sample, the carbonyl (C=O) contribution remained evident, as indicated by the clear presence of the 1711 cm^–1^ peak in the FTIR spectrum (Figure S1). In contrast, this peak was negligible for rGO2, confirming an effective reduction. As expected, NaBH_4_ efficiently reduced carbonyl (C=O) species while exhibiting only moderate reactivity toward carboxylic acids and no reactivity toward hydroxyl groups. Thus, in rGO2, the carbonyl groups were reduced, while the hydroxyl groups were slightly increased, probably due to the conversion of carbonyls to hydroxyls. The COOH peak overlaps with the C–O signal in the GO sample, making its contribution challenging to deconvolute accurately, while it increased substantially for both reduced samples, a finding supported by FTIR analysis.

ICP-MS was employed to simultaneously quantify trace amounts of paramagnetic manganese and iron impurities. While the former is mainly related to GO synthesis, the origin of the latter contamination has been traced back to the raw graphite material used in sample preparation. Understanding and quantifying iron level is critical, as it can influence both the structural properties and potential applications of the synthesized materials.^38^ Manganese and iron contaminations are reported in Table 3.

**Table 3:** ICP-MS Results for Graphite, GO, rGO1, and rGO2 Samples. Micromolar Metal Concentrations Were Calculated at the Highest MRI Sample Concentration Used (1 mg/mL)

Sample	Mn (ppm)	Fe (ppm)	Mn (μM)	Fe (μM)
**Graphite**	66 (±1)	4079 (±462)	1.20 (±0.02)	73.0 (±8.3)
**GO**	76 (±2)	789 (±27)	1.38 (±0.02)	14.1 (±0.48)
**rGO1**	57 (±7)	1040 (±20)	1.04 (±0.13)	18.6 (±0.4)
**rGO2**	3.2 (±0.2)	38 (±1)	0.058 (±0.003)	0.68 (±0.02)

The manganese level in the initial commercial graphite was found to be 66 ppm, slightly increasing to 76 ppm in the synthesized GO (permanganate was used as the oxidant reagent, but an accurate cleaning protocol was adopted^24^). Reduction treatments significantly decreased manganese concentrations: 57 ppm in rGO1 and a remarkable 3.2 ppm in rGO2. Iron contamination was notably higher across all samples. The starting commercial graphite exhibited a substantial iron concentration of 4079 ppm, which was reduced to 789 ppm in the GO. We measured a larger Fe concentration of 1040 ppm in thermally reduced rGO1. Excluding iron contaminations during the procedure, the higher Fe concentration could be related to the mineralization step during ICP-MS sample preparation: a more efficient mineralization of the thermally treated sample may have led to a higher Fe concentration. In contrast, the chemical reduction treatment in rGO2 resulted in a significant decrease (20-fold with respect to GO) in the iron content. The chemically reduced sample undergoes a solution-phase reaction, followed by an additional washing step of the resulting solid, which likely leads to a more effective removal of residual impurities and therefore a cleaner sample. Consistent with Poimenova et al.,^38^ our findings indicate that metallic impurities in GO-derived materials are strongly influenced by both the synthesis route and subsequent treatment steps, with chemical purification/reduction proving particularly effective in lowering residual metal content, thereby supporting our hypothesis that our protocol helps minimize contamination. These results underscore the importance of carefully controlling and understanding elemental contamination in graphite-derived materials as residual metals can significantly influence the physicochemical properties and potential applications of the resulting nanomaterials. Furthermore, they highlight the need for a precise description of the processing history of the materials prior to metal quantification, together with the use of robust analytical techniques, such as ICP, to accurately determine the nature and extent of residual elemental impurities. However, elemental analysis alone does not provide information about the electronic state or magnetic activity of these impurities. To identify the paramagnetic species effectively contributing to the magnetic response of the materials, we complemented the ICP measurements with EPR spectroscopy.

EPR spectroscopy detects paramagnetic species, i.e., atomic or molecular centers with unpaired electron spins. We used X-band CW-EPR to detect the paramagnetic species present in our samples, focusing on paramagnetic carbon defects, Mn­(II), and Fe­(III) metals. Figure 3 shows the RT EPR spectra of the highly purified GO, rGO1, and rGO2 nanosheets. Panel (a) presents the full spectral range, highlighting the absence of spectral features at a low field. The three main signals, attributed to localized paramagnetic carbon defect centers,^22,39,40^ are scaled to the same GO peak height. For this purpose, a scaling factor of 0.2 was applied to the rGO1 spectrum and a factor of 0.6 to the rGO2 spectrum. Panel (b) provides an enlarged view of the carbon defects peak, centered at approximately 353 mT (the three spectra are shown at their actual intensities, with no scaling factors applied). This comparison illustrates the effect of different reduction procedures on the carbon defects in the material. Additional spectral features are visible around the main peak in the GO EPR spectrum (Figure 3a, black line). These features correspond clearly to the six-line hyperfine pattern characteristic of manganese­(II).^23,41^ The Mn­(II) signal was also observed in the thermally reduced rGO1 sample (Figure 3c, red line), where the simulated spectrum (black dotted line) is superimposed. The Mn­(II) signal was not detected in the rGO2 sample (Figure 3a, blue line). Figure 3d shows the single narrow peak observed in the rGO2 sample in more detail as well as the simulated Lorentzian line. At RT, no other signals were present in the 120–200 mT region, where the iron EPR signal typically appears.

**Figure 3:**
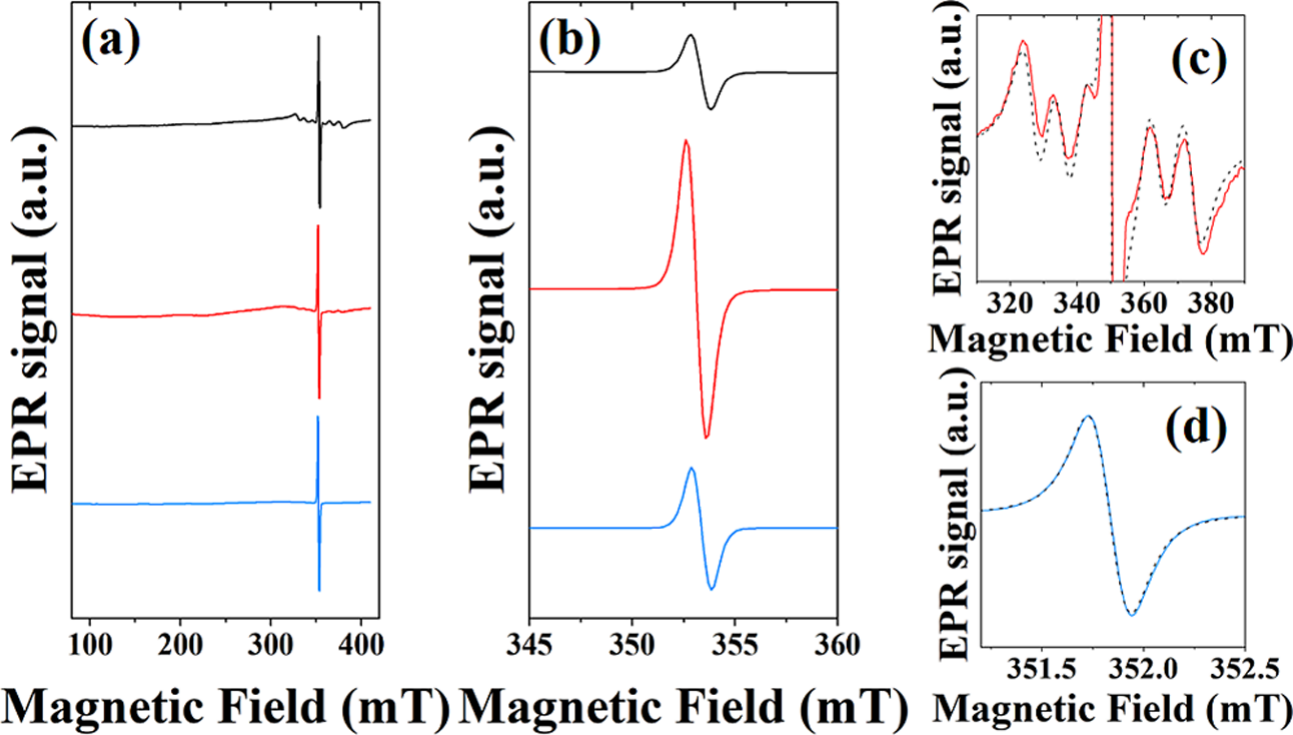
RT EPR experimental spectra of solid-state GO (black), rGO1 (red), and rGO2 (blue). (a) The spectra are scaled to the same peak-to-peak height of the carbon defect line. (b) Carbon defect spectra without scaling factors. (c) Detail of the rGO1 spectrum (red line), along with the simulated spectrum (black dotted line), in the 310–390 mT field range. (d) Detail of the rGO2 spectrum (blue line), along with the simulated spectrum (black dotted line), in the 351.2–352.5 mT field range.

The simulated Mn­(II) and carbon defect spectral features were superimposed as dotted lines in the two rGO samples. The agreement between simulation and experiment was excellent for the rGO2 spectra, dominated by the single Lorentzian line of carbon defects (Figure 3d). The fit was less accurate for the more complex Mn­(II) hyperfine structure visible in rGO1 (Figure 3c). The fit with the simulated Lorentzian curve provided a line width of 0.16 mT in the GO and rGO1 samples and 0.21 mT in the rGO2 sample. These line widths are within the range reported by other authors.^42^ The simulation of the rGO2 spectrum, with no superimposed Mn­(II) signal, should be considered as the most accurate for the carbon center defects (Figure 3d).^43^ The slight broadening of the signal in the most reduced sample is closer to the response of a conductive sample, as corroborated by the literature.^43^ The Mn­(II) signal from the GO and rGO1 spectra shows a line width of 6.40 mT, which was estimated from the simulated spectra using Gaussian curves.

To enhance the signal-to-noise ratio and detect other metallic impurities that are not observable at RT, EPR measurements were also performed at 100 K. As shown in Figure 4a, the full EPR spectra at 100 K reveal, as for the RT ones, the presence of the carbon defect signals in all samples and the Mn­(II) signal in the GO and rGO1 samples. In panel (a), the spectra are scaled to the same peak-to-peak height, applying a scaling factor of 0.2 to the rGO1 spectrum and of 2.8 to the rGO2 spectrum. Notably, a broad signal corresponding to high-spin Fe­(III) (g value of 4.2602) was observed in the low-field region for all three samples. In Figure 4b, the iron features at low field are shown without any scaling factor.

**Figure 4:**
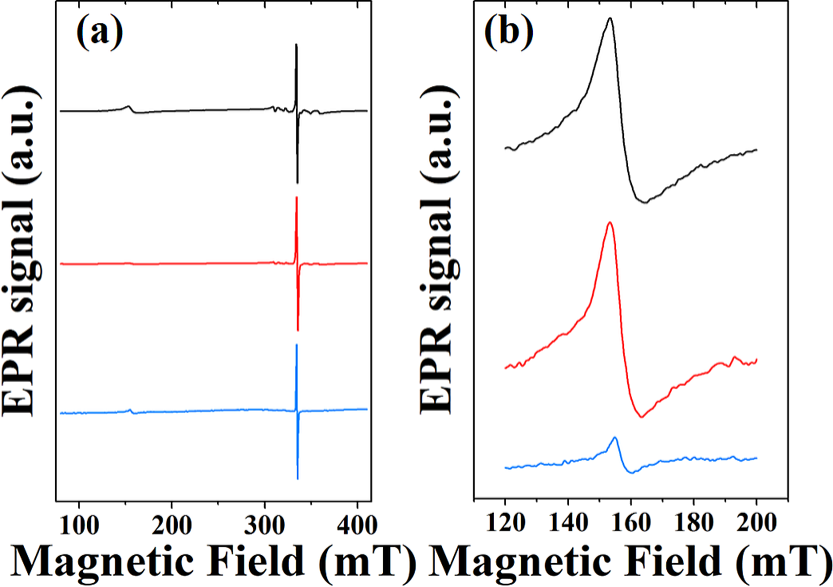
Low-temperature (100 K) X-band CW EPR spectra of GO (black), rGO1 (red), and rGO2 (blue): (a) full range spectra are scaled to the same peak-to-peak height and (b) enlarged EPR spectra without scaling factors, in the 120–200 mT field region of the high-spin Fe­(III) component.

The spectral area for the different types of paramagnetic centers were computed. These values do not convert into an absolute number of spins per gram because (i) the modulation amplitude used to detect carbon defects and Mn in the same spectra was not optimal for simultaneous quantification; (ii) there was no reference sample to compare with; and (iii) the Fe signal was detected at a much lower temperature. Considering the above limitations, in the GO sample, we estimate 2.6 × 10^11^ a.u./g/[S­(S+1)] for carbon defects, 3.8 × 10^11^ a.u./g/[S­(S+1)] for Mn­(II), and 288.3 au for the spectral area of Fe­(III). Table 4 shows the relative amounts of the paramagnetic centers in the three graphene samples with respect to the corresponding GO values (these factors cannot be directly compared to the scaling factors of Figure 3a because the spectra were acquired with different parameters). Data indicate a sharp increase in carbon defects for the thermally reduced sample, rGO1, milder for rGO2. This observation can be ascribed to the different amounts of carbon defects retained in the graphene structure after the removal of oxygenated groups in the different reduction processes, as suggested by the XPS C–O data (Table 2).

**Table 4:** Relative Amounts of Carbon Defects, Mn­(II), and Fe­(III) Paramagnetic Centers from the Double Integral (DI) of the Simulated EPR Peak Spectral Area, Corrected for Sample Mass, Spin Multiplicity, and Normalized to the Corresponding GO Signal^a^

Sample	DI (a.u.)* Carbon defects	DI (a.u.)** Mn(II)	Spectral area (a.u.)*** Fe(III)
**GO**	1.00	1.00	1.00
**rGO1**	4.80	2.40	0.90
**rGO2**	1.71	N.D.^#^	0.05

a The relative amounts of iron centers, associated with the Fe­(III) peak, were determined from the experimental spectral areas of the 100 K EPR signals. EPR acquisition parameters (*) 0.1 mT modulation, 15 mW, and 20 mT field range; (**) 1 mT modulation, 1 mW, and 140 mT field range; (***) as in Figure 4 for Fe­(III). # N.D. stands for not detectable.

Iron 100 K EPR data, corresponding to the Fe­(III) peak, with acquisition parameters as in Figure 4a, are presented in the third column of Table 4. We observe a trend analogous to that from ICP-MS: the relative abundance of Fe­(III) slightly changes following the thermal treatment (rGO1), while it has a significant decrease with the chemical treatment (rGO2).

Relaxation-weighted MR images recorded at 1.0 T for GO, rGO1, and rGO2 in aqueous solutions demonstrated that all of the samples have a remarkable effect on the T1 and T2 relaxation, with a clear linear correlation between the sample concentrations and the longitudinal (Figure 5a) and transverse (Figure 5b) relaxation rates.

**Figure 5:**
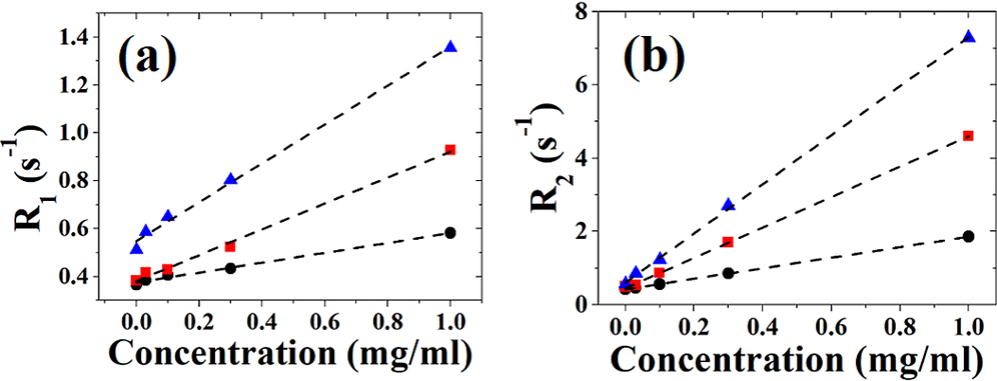
RT (22 °C) 1.0 T MRI data for (a) longitudinal (R1 = 1/T1) and (b) transverse (R2 = 1/T2) relaxation rates of Milli-Q water solution with GO (black circles), rGO1 (red squares), and rGO2 (blue triangles) with different sample concentrations (0; 0.01; 0.1; 0.3; and 1.0 mg/mL).

The longitudinal (*r*_1_) and transverse (*r*_2_) relaxivity values (mg/mL s)^−1^ and the coefficients of determination (*R* ^2^) are reported in Table 5. The data show a clear trend of relaxivities increasing with the level of reduction, reflecting changes in their MR contrast-enhancement capabilities. As reported in Table 5, all *R* ^2^ values are close to 1 for the samples, indicating generally good linear fits of the relaxation rate versus concentration and confirming the reliability of the 1.0 T measurements. The linear trend for GO *r*_1_, although showing greater scatter and lower *R* ^2^ value, remains statistically significant (*p* < 0.05), albeit with a larger relative error than for rGO1 and rGO2.

**Table 5:** RT (22 °C) 1.0 T MRI Data for Longitudinal (*r*_1_) and Transverse (*r*_2_) Relaxivities of Milli-Q Water Solution with GO, rGO1, and rGO2. For Each Sample, the Regression Coefficient of the Linear Fit (*R* ^2^) Is Reported^a^

Sample	*r*_1_ (mg/mL s)^–1^	*R* ^2^	*r*_2_ (mg/mL s)^–1^	*R* ^2^
**GO**	0.21 (±0.08)	0.850	1.44 (±0.09)	0.9990
**rGO1**	0.54 (±0.11)	0.996	4.15 (±0.25)	0.9997
**rGO2**	0.81 (±0.15)	0.995	6.70 (±0.41)	0.9995

a Increasing the reduction degree (from GO to rGO1 to rGO2) significantly shortens both relaxation times, enhancing *r*_1_ and *r*_2_ relaxivities, with a slightly more pronounced effect for *r*_2_ (*a* factor of 4.6 for rGO2 relative to GO) than for *r*_1_ (*a* factor of 3.9).

This trend provides strong evidence that chemical and thermal reduction of pristine GO improves MRI contrast properties by restoring conjugated networks and modifying the surface chemistry. It is also important to note that the above results cannot be ascribed to the presence of metallic paramagnetic contaminants.

To provide a quantitative upper bound on the possible contribution of residual metals, the ICP-MS concentrations (Table 3) were converted to molar concentrations assuming a dispersion concentration of 1 mg/mL, corresponding to the highest concentration used in the MRI experiments. The resulting concentrations were [Mn] = 1.38, 1.04, and 0.058 μM and [Fe] = 14.1, 18.6, and 0.68 μM for GO, rGO1, and rGO2, respectively. These values represent conservative upper-bound estimates, as they assume that all metal impurities are completely dissolved, fully accessible to water, and present as free paramagnetic ions in solution, which corresponds to the most favorable scenario for maximizing relaxivity contributions. Using these concentrations, the maximum possible relaxation rate enhancements at 1 mg/mL were estimated according to Δ*R*__max_ = [Mn]·r­(Mn^2+^) + [Fe]·r­(Fe^3+^). Upper-bound literature relaxivities for free Mn^2+^ (*r*_1_ = 5.7 mM^–1^ s^–1^, *r*_2_ = 57 mM^–1^ s^–1^)^24^ and Fe^3+^ (*r*_1_ = 1.5 mM^–1^ s^–1^, *r*_2_ = 4.0 mM^–1^ s^–1^)^44^ ions in an aqueous solution at 1.0 T were used for the calculation. Under these assumption, the maximum possible metal-induced relaxation enhancements were estimated to be Δ*R*_1_ ≤ 0.029 s^–1^ and Δ*R*_2_ ≤ 0.128 s^–1^, respectively, across all samples. These values represent at most 14% and 9% of GO Δ*R*_1_,_2_, and fall below 0.2% for rGO2, the sample with the highest relaxivity. This quantitative upper-bound analysis therefore demonstrates that paramagnetic metal impurities make a negligible contribution to the observed relaxivity enhancement upon GO reduction.

To investigate the connection between MRI relaxivities and samples’ structure, we reported in Figure 6 the longitudinal (violet circles) and transverse (green squares) relaxivities vs the XPS C/O ratio from Table 1 (Figure 6a) and the XPS C–C/C=C (%) from Table 2 (Figure 6b). The coefficients of determination (*R* ^2^) for the linear correlation of *r*_1_ and *r*_2_ vs XPS C/O ratio were 0.9967 and 0.9997, respectively, and 0.9608 and 0.9748 for the XPS C–C/C=C (%) case. In both plots and for both relaxivities, we observe an excellent linear correlation. The results are summarized in Table 6.

**Figure 6:**
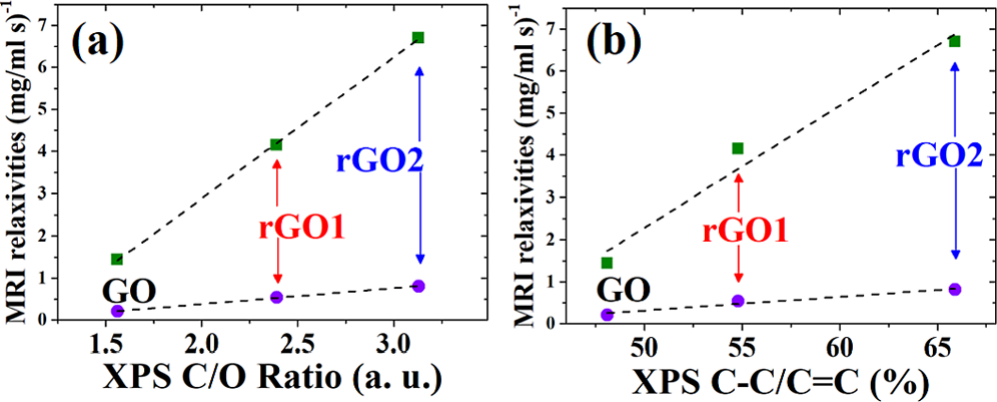
Longitudinal (*r*_1_, violet circles) and transverse (*r*_2_, green squares) relaxivities versus (a) the XPS C/O ratio and (b) XPS C/C–C=C (%) for the GO, rGO1, and rGO2 samples.

**Table 6:** Coefficients of Determination (*R* ^2^) for the Linear Correlation among the MRI Relaxivities and the XPS Survey Data (Figure 6) of the GO, rGO1, and rGO2 Samples

	*R* ^2^
*r*_1_vs C/O **ratio** (**from XPS survey data**)	0.9967
*r*_2_vs C/O **ratio** (**from XPS survey data**)	0.9997
*r*_1_vs C–C/C=**C %** (**from XPS C 1s data**)	0.9608
*r*_2_vs C–C/C=**C %** (**from XPS C 1s data**)	0.9748

## 4 Conclusions

This study demonstrates, for the first time, a clear and progressive enhancement of MRI relaxivities with an increasing degree of GO reduction. We devoted many efforts to accurately cleaning the samples to limit the confounding effects from paramagnetic contaminants. EPR spectroscopy and ICP-MS confirmed that neither Mn­(II) nor Fe­(III) impurities are responsible for the observed relaxivity trend. Instead, strong linear correlations between *r*_1_ and *r*_2_ and the XPS-derived C/O ratio and C–C/C=C content indicate that carbon structural defects govern MRI contrast performance.

Although a detailed identification of these carbon defects lies beyond the scope of the present EPR/MRI study, several considerations can be made. The quantification of DI carbon defects qualitatively follows the same trend as the XPS-detected C–O bonds: both increase from GO to rGO1 and decrease to intermediate values in rGO2 (Tables 2 and 4). This suggests that the higher defect density observed in rGO1 may originate from the partial opening of epoxy rings and their conversion to hydroxyl groups. However, this hypothesis requires a further targeted investigation.

MRI relaxivities were measured in an aqueous solution, whereas defect concentrations (as well as C–O bond content) were determined in the solid state. While relaxivities are governed by the presence of paramagnetic centers, they are also strongly influenced by (i) the dynamics of water molecules within the hydration layer surrounding GO/rGO sheets and (ii) quenching mechanisms occurring in solution. Notably, no direct correlation is observed between DI defect concentration and relaxivities: the former is highest in rGO1, whereas relaxivities reach their maximum in rGO2 (Tables 3 and 4).

The chemically reduced rGO2 sample exhibits the highest relaxivities, approximately 3.9-fold (*r*_1_) and 4.6-fold (*r*_2_) higher than those of pristine GO. These findings provide a rational basis for engineering rGO-based MRI CAs through controlled reduction, with water molecule dynamics and defect-related quenching effects in solution warranting further investigation.

## Supplementary Material



## Notes

Synthetic procedures for GO and rGO samples, as well as Fourier-transform infrared (FTIR) spectroscopy, RS, DSC, thermogravimetric analysis (TGA), XPS survey analyses, and Scanning Electron Microscopy images (PDF)
