# Supplementary material for: Reduction-Driven Modulation of Relaxivity in Graphene Oxide Derivatives
Source: ACS Omega. 2026 Jul 31;11(31):46701–10. doi: 10.1021/acsomega.6c04502 (PMC13470764; doi:10.1021/acsomega.6c04502)
Supplement: Supplementary file 1 [file ao6c04502_si_001.pdf]

Supporting Information file

Reduction-Driven Modulation of Relaxivity in Graphene Oxide Derivatives

Giulia Fioravanti[a], Laura Torrieri Di Tullio[b], Salvatore Mamone[c], Paola Fattibene[b], Marcello Alecci[c], Silvia Colacicchi[c], and Angelo Galante[c, d, e]**

[a] Department of Physical and Chemical Sciences, University of L'Aquila, 67100 L'Aquila, Italy.

[b] Istituto Superiore di Sanità, Viale Regina Elena 299, 00161 Rome, Italy.

[c] Department of Life, Health and Environmental Sciences, University of L'Aquila, 67100 L'Aquila, Italy.

[d] National Institute for Nuclear Physics (INFN), Gran Sasso National Laboratory (LNGS), 67100 L'Aquila, Italy.

[e] CNR-SPIN, c/o Department of Physical and Chemical Sciences, 67100 L'Aquila, Italy.

*Corresponding Authors: Giulia Fioravanti, Email: giulia.fioravanti@univaq.it; Angelo Galante, Email: angelo.galante@univaq.it

Index

Materials and Methods

1. Synthesis of the GO and rGO samples

2. Characterization of GO and rGO samples

2.1 Fourier-Transform Infrared Spectroscopy (FTIR)

2.2 Raman Spectroscopy (RS)

2.3 Differential Scanning Calorimetry (DSC)

2.4 Thermogravimetric Analysis (TGA)

2.5 X-Ray Photoelectron Spectroscopy (XPS)

2.6 Scanning Electron Microscopy (SEM)

Materials and Methods

1. Synthesis of the GO/rGO samples

Graphene oxide (GO) was synthesized using a modified Hummers method[1]. GO was prepared from graphite flakes, with an average particle size of 150 micron, purchased from Sigma-Aldrich (graphite, quality level 100, product N. 332461). Concentrated sulfuric acid (H_2SO_4, 96%, product N. 30743), sodium nitrate ($NaNO_3$, 99%, product N. 221341), potassium permanganate ($KMnO_4$, >99%, product N. 60458), hydrogen peroxide solution (H_2O_2, 30%, product N. 95294), hydrochloric acid (HCl, 37%, product N. 30721), sodium borohydride ($NaBH_4$, 96%, product N. 71320) were purchased from Sigma Aldrich (St Louis, MO). All the aqueous solutions were prepared using ultrapure MilliQ distilled water (Millipore, Bedford, MA, USA).

Graphite (5 g) and sodium nitrate (3.8 g) were placed into a beaker in a salt/ice bath. Subsequently, 375 ml of concentrated sulfuric acid was added. The reaction mixture was kept in continuous agitation by a mechanical stirrer. After the mixture had become homogeneous, 25 g of potassium permanganate were slowly added. The solution was kept under stirring for 5 days at room temperature. After 5 days, a 5% H_2SO_4 aqueous solution (700 ml) was poured through a funnel, and H_2O_2 (30 wt%) was added drop by drop to remove the potassium permanganate, and the suspension was thus stirred for another 2 hr. In order to obtain a clean product, the mixture was diluted with 5% H_2SO_4 (2 l) and left to settle for 1 day. Inorganic impurities were removed through successive centrifuges removing the

supernatant. The solid part was washed/centrifuged at 4000 rpm for 10 min with a 5% aqueous solution of H_2SO_4 and H_2O_2 at 0.3% (12 times), then 4% HCl (3 times), deionized water (8 times), and finally MilliQ water (2 times), removing the supernatant after each passage. The pH of the dispersion was monitored until it reached 6–7. Finally, the GO was transferred to acetone and dried at 50 °C for 24 hours, obtaining 4.8 g of powder.

Thermal reduced GO samples (rGO1) were prepared by thermal treatment of the GO in a laboratory oven, starting from dried GO powder, kept at 80 °C for 24 hours. Chemically reduced GO samples (rGO2) were obtained following well-known procedures[2] from a water suspension of GO (100 ml at 0.6 mg/ml) mixed with 50 ml 1 M water solution of $NaBH_4$ under reflux for 1 hour. Then the reaction mixture was cooled to RT, washed several times in ultrapure milliQ water (by centrifugation) till the pH of the filtrate stabilized to ca. 7-8, and then dried at 60 °C.

2. GO/rGO characterization

The GO/rGO samples were characterized by Fourier-Transform Infrared Spectroscopy (FTIR), Raman Spectroscopy (RS), Differential Scanning Calorimetry (DSC), Thermogravimetric Analysis (TGA), X-Ray Photoelectron Spectroscopy (XPS) as described below. Inductively Coupled Plasma-Mass Spectrometry (ICP-MS), Electron Paramagnetic Resonance (EPR) and 1.0 T Magnetic Resonance Imaging (MRI) were also used, as reported in the main text.

2.1 Fourier-Transform Infrared Spectroscopy (FTIR)

Room temperature (RT) FTIR spectra were performed at 4000-400 cm^{-1} wavelength on a Perkin Elmer spectrophotometer Spectrum Two, equipped with a reflectance module (ATR) at 4 cm^{-1} spectral resolution, with the accumulation of 16 repeated scans. The GO powder was completely dried in an oven for at least 24 hours at 60 °C, and then directly characterized. The FTIR spectra of the GO, rGO1 and rGO2 samples were performed in the range of 4000-400 cm^{-1} for the identification of functional groups, as reported in Figure S1. Analysing the data, we observed the presence of different absorption peaks, according to the spectra reported in the literature.

The GO FT infrared spectrum, Figure S1-a (black line), evidenced the -OH stretching vibration at about 3420 cm^{-1}. The vibrational bands at 2923 cm^{-1} and 2854 cm^{-1} are attributed to the stretching vibrations of CH and CH_2 groups associated with sp^3 carbon. The absorption band at 1725 cm^{-1} corresponds to stretching vibrations of C=O from carbonyl or conjugated carbonyl groups. The absorption peak at 1580 cm^{-1} is assigned to the C=C (aromatics) stretching, partially overlapped by the strong bending band of water observed at 1620 cm^{-1}. The absorption peaks at about 1365 cm^{-1}, 1278 cm^{-1}, and 1041 cm^{-1} are assigned to C-OH bending from tertiary alcohols, C-OH bending from carboxyl groups, and C-OH stretching from tertiary alcohols, respectively. The shoulders at 986 cm-1 and 870 cm^{-1} are attributed to the C-O-C stretching from epoxy or ether groups, respectively. These results agree with the literature[3, 4]. The low intensity signals at 1411 cm^{-1} and 1227 cm^{-1}, respectively, are caused by asymmetric and symmetric vibrations of the S=O bonds of covalently bound sulfates, due to the GO synthesis Hummers' method, which are minimized by the purification protocols, which hydrolize the sulfate groups. The signals at 1411 cm^{-1} and 1227 cm^{-1}, although not intense, can be attributed to the asymmetric and symmetric vibrations of the S=O bonds of covalently bound sulphate groups, typically introduced by the Hummers' method. However, their presence is minimized through rigorous purification protocols that hydrolyze and remove the residual sulphate groups, as reported in our previous work[5].

In the thermally reduced rGO1 sample (Figure S1-b, red line) the C=O stretching peak is shifted to 1711 cm^{-1}, while the C=C signal is no longer obscured by the OH bending contribution from water and is more prominent at 1578 cm^{-1}. The reduction process was mild, preserving most of the oxygenated functionalities on the graphenic skeleton. The C-OH stretching signal from tertiary alcohols at 1035 cm^{-1} remains strong. The shoulder at 980 cm^{-1}, related to the C-O-C stretching from epoxy groups, is less pronounced, while the low-intensity band of the epoxide at 870 cm^{-1} is still present.

In the chemically reduced rGO2 sample (Figure S1-c , blue line) the sodium borohydride reduced the C=O species (disappearing of the 1720 cm^{-1} peak), while the C=C stretching signal at 1590 cm-1 is

highlighted. Vibrations at 1640 cm^{-1}, 1380 cm^{-1}, and 1240 cm^{-1} are typical of carboxylate asymmetric and symmetric stretching, and also OH bending. Hydroxyl groups were retained as evidenced by the strong signal at 1337 cm^{-1} related to the bending of tertiary alcohol, as well as epoxy groups, showing residual peaks at 990 cm^{-1} and 820 cm^{-1}.

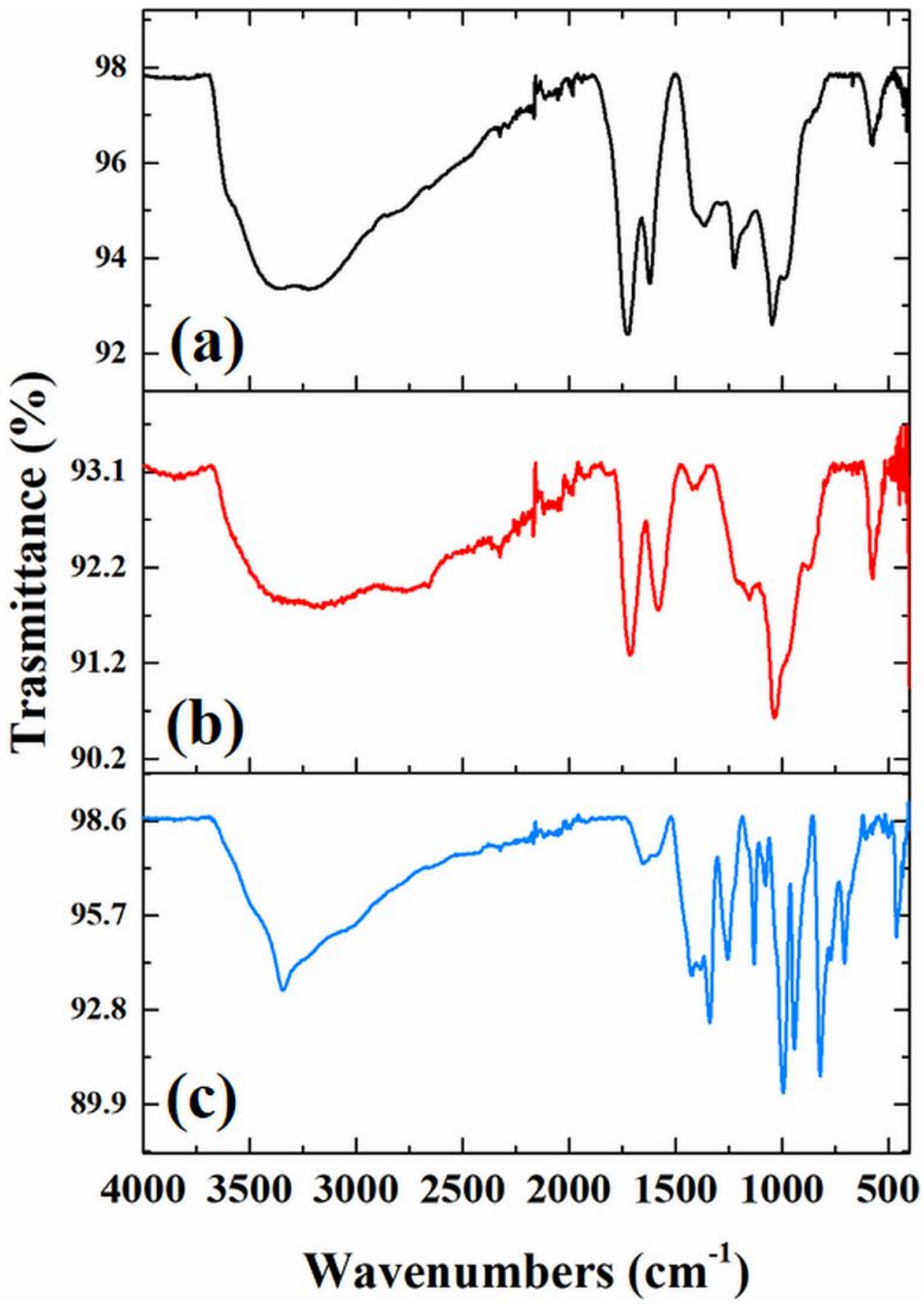

Figure S1. FTIR spectra of (a) GO (black line), (b) rGO1 (red line) and (c) rGO2 (blue line).

2.2 Raman Spectroscopy (RS)

GO and rGO flakes dispersed in Milli-Q water were deposited, by drop-casting, from a dilute aqueous solution (0.3 mg/ml, volume of 15 μl) of the material on a silicon substrate (SiO_2, oxide thickness 270 nm). RS was performed using a LabRAM confocal microscope (Horiba-Jobin Yvon- Japan, operating in the back-scattering configuration), equipped with a diode-pumped solid-state (DPSS)

laser (excitation wavelength = 532 nm) and with a He-Ne gas laser (excitation wavelength = 633 nm), 1 µm spatial resolution, and about 2 cm^{-1} spectral resolution.

Raman spectra of GO, rGO1 and rGO2 are reported in Figure S2. The D vibrational band, due to defects and disorders in the carbon lattice, can be seen at 1348 cm^{-1}, 1343 cm^{-1} and 1356 cm^{-1} for GO, rGO1, and rGO2, respectively. The G vibration band, characteristic of graphitic domains, was found for the three samples at 1598 cm^{-1}, 1585 cm^{-1} and 1606 cm^{-1}, respectively. The I_D/I_G ratio was 1.08 in GO, 1.04 in rGO1 and 0.91 in rGO2, with a progressive decrease indicating partial restoration of sp^2 carbons, with the chemical reduction protocol showing greater recovery. The RS results also reveals broad 2D, D+D' and 2D' bands, shifted to higher wavenumbers than pristine monolayer graphene, confirming multilayer graphene structures in all samples.

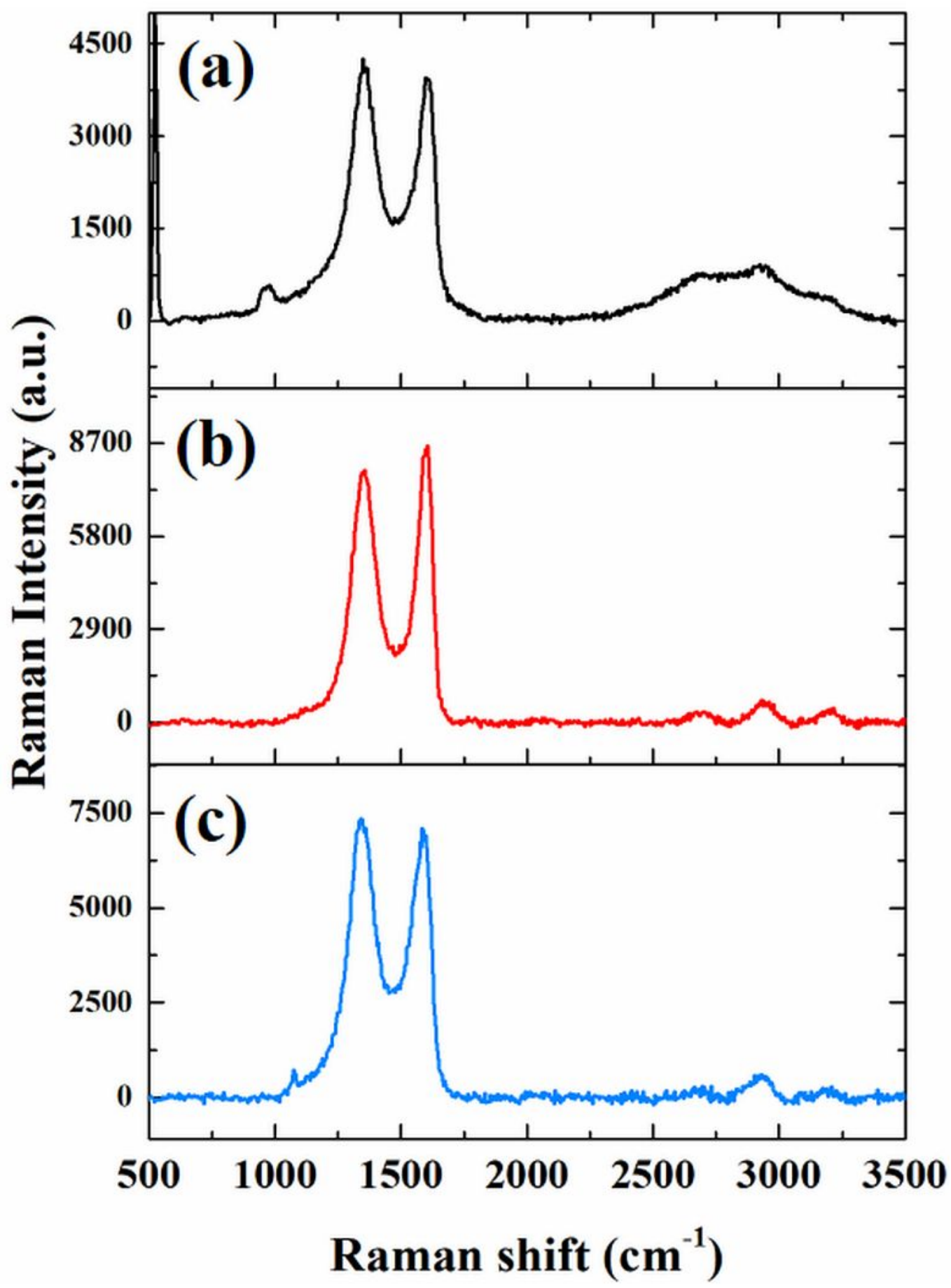

Figure S2. Raman spectra of the (a) GO (black line), (b) rGO1 (red line) and (c) rGO2 (blue line) samples.

2.3 Differential Scanning Calorimetry (DSC)

DSC measurements were carried out with a Mettler Toledo DSC 3 calorimeter (Mettler-Toledo International Inc., Columbus, OH, USA). GO, rGO1 and rGO2 samples were directly inserted into Aluminum pans of 40 μL. A single heating scan was performed from 30°C to 300°C, heating rate of 10°C/min, in N_2 atmosphere. Under the experimental conditions, reproducible thermal recordings were obtained. The uncertainty on temperatures was ±0.1 °C, and that on ΔH was ±0.5 kJ/mol.

In Figure S3 are reported the thermograms of GO, rGO1 and rGO2.

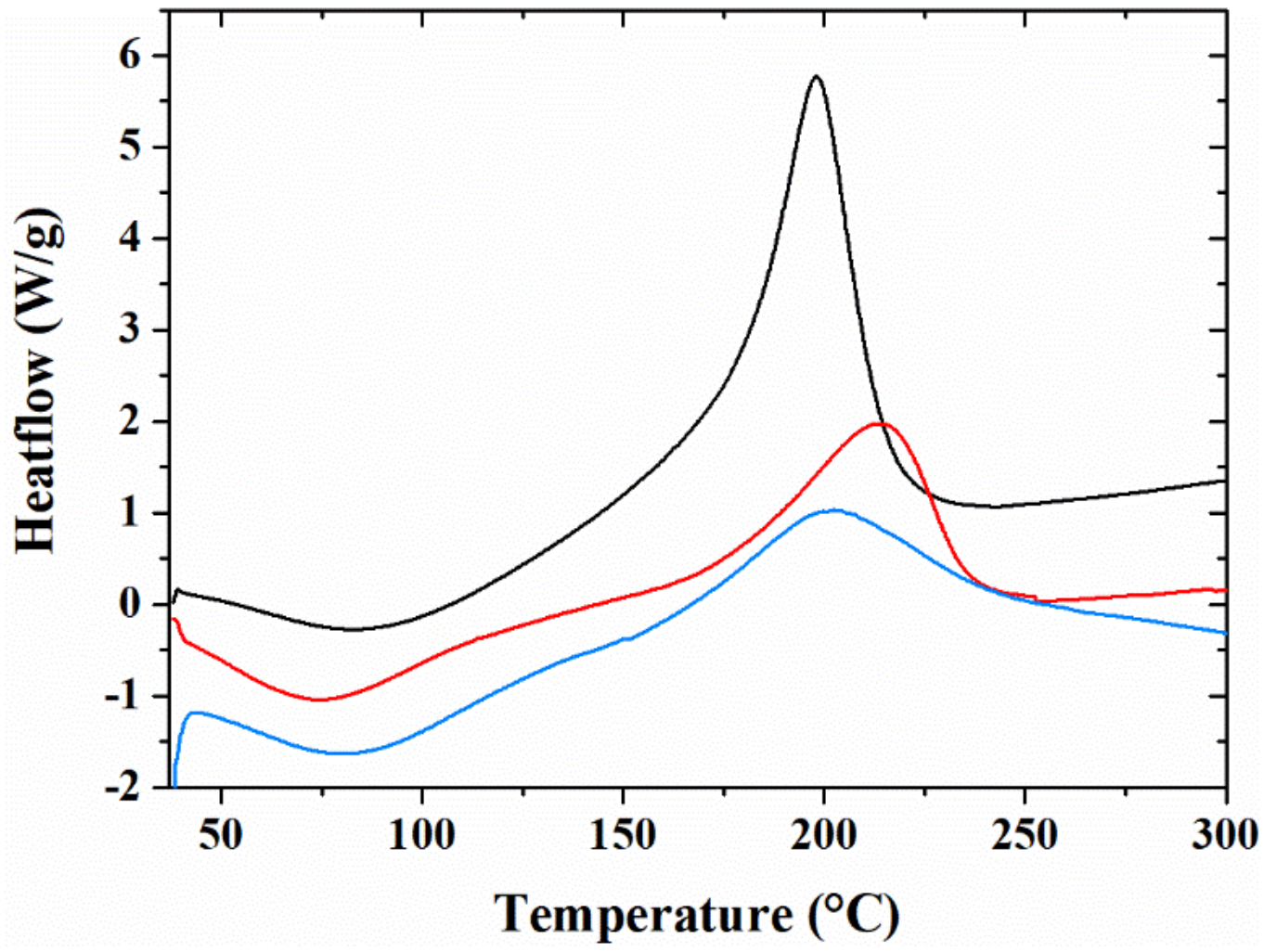

Figure S3. DSC thermograms (exothermic up) of GO (black line), rGO1 (red line), and rGO2 (blue line).

The DSC analysis of GO and reduced samples reveals distinct thermal behaviour indicative of their structural transformations. GO exhibits a broad endothermic peak centred around 90 °C, attributed to the removal of moisture and labile oxygenated functional groups. This is followed by a pronounced exothermic peak at 198 °C, corresponding to the decomposition and removal of the more stable oxygenated groups, with an enthalpy change of 758 J/g (Table S1). Upon reduction, the exothermic peak intensity decreases progressively in the order GO > rGO1 > rGO2, consistent with the extent of oxygen removal during the reduction process. Thermal reduction is milder than chemical reduction, as evidenced by the higher enthalpy for rGO1 (431 J/g) compared to rGO2 (146 J/g), showing that some oxygen groups persist after thermal treatment. Peak temperature and relative normalized enthalpies of the GO, rGO1, and rGO2 samples are reported in Table S1.

Table S1. Peak temperature and relatives normalized enthalpies of GO, rGO1 and rGO2 samples.

Sample	Temperature (°C)	Enthalpy (J/g)	Temperature (°C)	Enthalpy (J/g)
**GO**	89	-243	198	758
**rGO1**	75	-181	213	431
**rGO2**	94	-222	201	146

2.4 Thermogravimetric Analysis (TGA)

TGA measurements were conducted with a Hitachi Nexta STA200RV instrument. The temperature range was from RT to 1000 °C, and the heating rate was 10 °C/min, in air atmosphere (flux 100 ml/min). The graphite, GO, rGO1, and rGO2 samples were inserted into Alumina open sample pans (GAA-1980) and directly weighted by the instrument (approximately 10 mg). TGA of the samples are reported in Figure S4. Percentage weight loss for GO, rGO1, rGO2, and graphite samples are reported in Table S2.

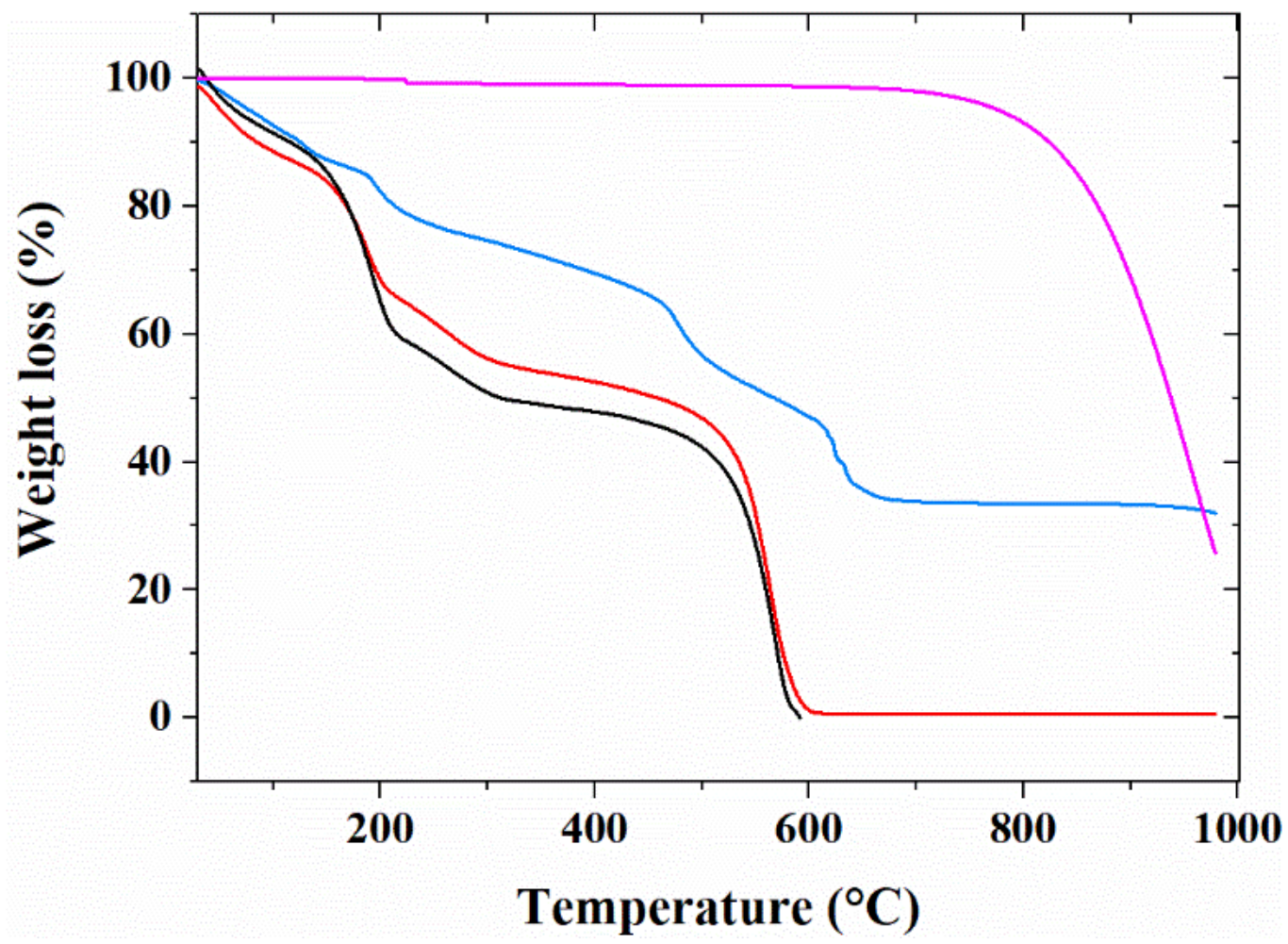

Figure S4. TGA curves of GO (black line), rGO1 (red line), rGO2 (blue line), and graphite (pink line).

Table S2. Weight percent loss for GO, rGO1, rGO2 and graphite samples. TGA was performed from room temperature up to 1000 °C in air.

	Weight Loss (%)			
**Sample**	**I (< 200°C)**	**I (500°C)**	**I (600°C)**	**I (800°C)**

**GO**	32.6	-	47.5	-
**rGO1**	22.0	-	51.7	-
**rGO2**	11.2	17.5	13.5	-
**Graphite**	-	-	-	> 80

The TGA profiles reveal the expected trend in thermal stability across GO, rGO, and graphite. The initial mass loss below 100 °C corresponds to the release of intercalated water and labile oxygen-containing functional groups, accompanied by a gradual weight decrease associated with continuous volatilization processes. A subsequent mass loss around 200 °C is attributed to the decomposition of remaining oxygenated groups, a characteristic feature of partially oxidized graphenic materials. GO displays the highest low-temperature mass loss (32.6%), consistent with its large content of labile oxygen functionalities and adsorbed water, whereas rGO1 and rGO2 show progressively lower losses (22.0% and 11.2%, respectively), reflecting a greater degree of reduction.

A major mass loss at approximately 600 °C corresponds to carbon combustion, marking the degradation of the carbon framework. rGO2 additionally exhibits noticeable mass losses at 500 °C and 600 °C (17.5% and 13.5%), suggesting the presence of more thermally stable oxygen species or persistent structural defects. In contrast, graphite remains highly stable up to high temperatures, showing significant mass loss (>80%) only near 800 °C, in line with its well-ordered and oxidation-resistant structure. Altogether, the samples follow the expected stability order: GO < rGO1 < rGO2 ≪ graphite.

2.5 X-Ray Photoelectron Spectroscopy (XPS)

XPS spectra were acquired under ultra-high vacuum (UHV) conditions using a PHI ESCA system equipped with an Mg Kα X-ray source (hν = 1253.6 eV) and a hemispherical electron energy analyzer. The peak areas were corrected using the appropriate Relative Sensitivity Factors (RSFs) prior to the determination of the atomic concentrations. The XPS spectra were analyzed by fitting the components with Voigt line shapes and Shirley-type backgrounds[6].

The samples were deposited on a Si substrate (1 cm^2, SiO_2, oxide thickness 270 nm). The substrate was previously sonicated 10 min with acetone (Sigma Aldrich product N. 24201, >99%), 10 min with

isopropyl alcohol (Sigma Aldrich product N. W292907, 99.7%), 10 min with diluted basis Piranha (1:3:4 of H_2O_2:NH_4OH:H_2O) (Hydrogen peroxide, Sigma Aldrich product N. 95294, 30%; Ammonia solution, Sigma Aldrich product N. 05002, 33%), and then finally rinsed with Milli-Q water. The GO/rGO samples were deposited by drop casting a dilute aqueous solution (1.0 mg/ml, volume of 20 µl) of the material on the substrate.

The XPS survey spectra (Figure S5) confirmed that carbon and oxygen are the main elements present in all samples. From XPS survey spectra, we calculated the total surface elemental composition, including C 1s, O 1s, N 1s, S 2p, and B 1s contributions and the C/O ratio, as reported in Table 1 of the main text. Very low amounts of N and S were detected in GO (approximately 1%), likely originating from the Hummers synthesis route, while 5.6% of B was observed in rGO2 due to the use of $NaBH_4$ during the reduction process. No Mn or Fe signals were detected, likely due to the very low concentrations (below 1%, which is the detection limit for XPS).

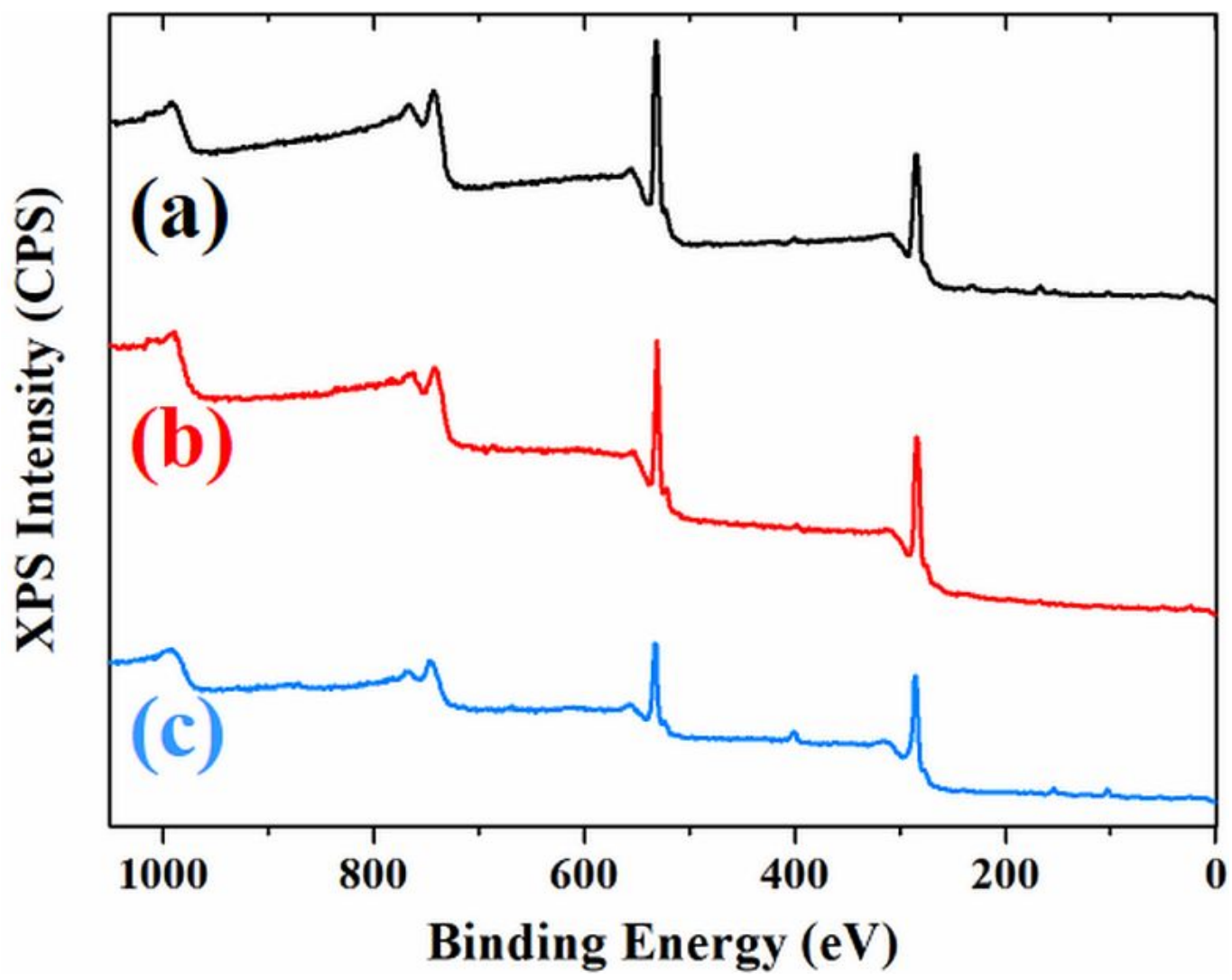

Figure S5. XPS survey of the (a) GO, (b) rGO1 and (c) rGO2 samples

2.6 Scanning Electron Microscopy (SEM)

Scanning electron microscopy (SEM) was performed using a Zeiss Gemini LEO 1530 system. Sonication of 1.0 mg/mL aqueous dispersions of GO/rGO samples was carried out using a Hielscher sonicator (model UP100H) for 1 hour, while maintaining the temperature below 30 °C using an ice

bath. Samples were deposited by spin coating on a Si substrate (1 cm^2, SiO_2, oxide thickness 270 nm).

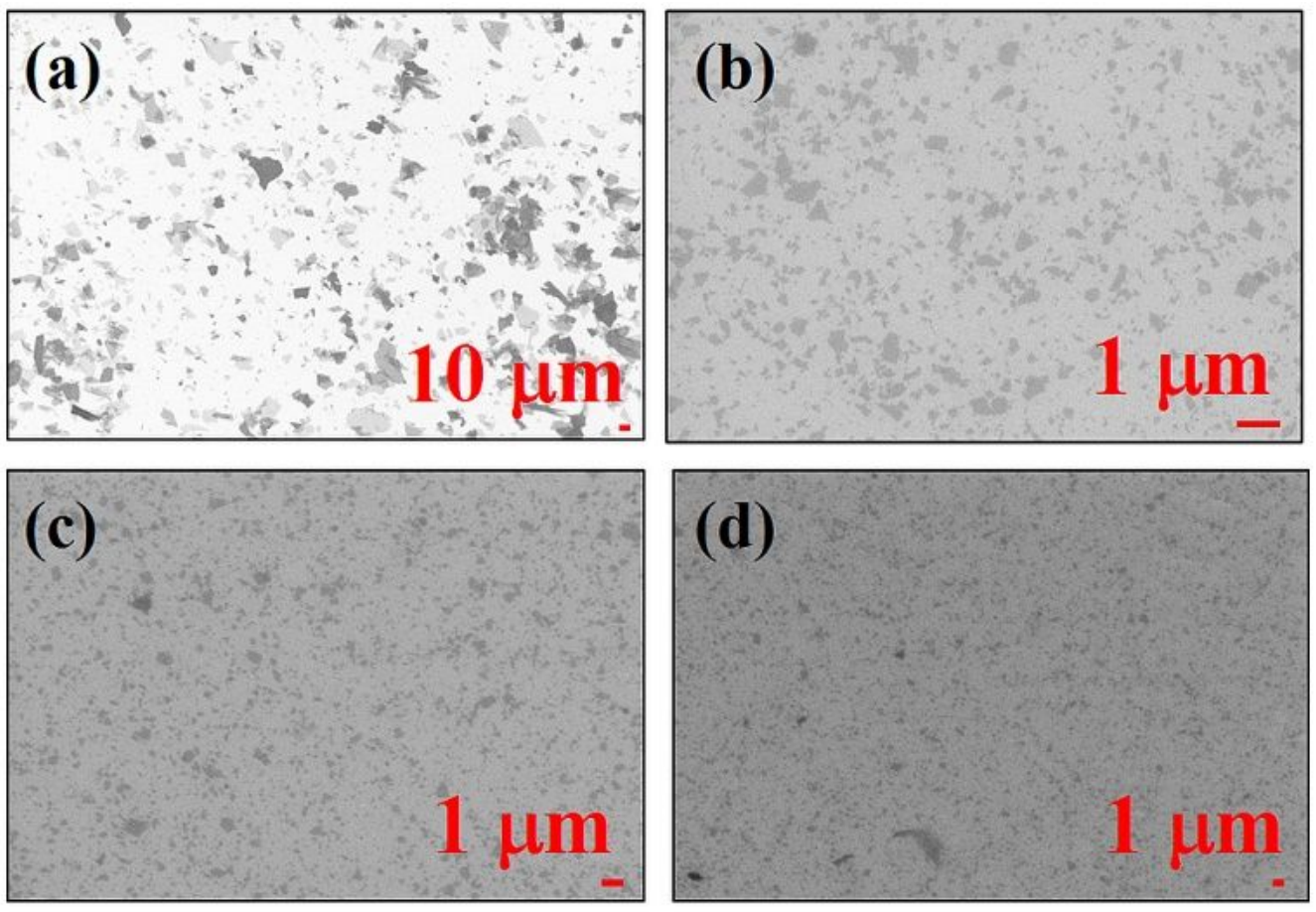

Figure S6. SEM images of (a) as-prepared GO, after 1h sonication of (b) GO, (c) rGO1 and (d) rGO2 samples.

References

1. E. Treossi, M. Melucci, A. Liscio, M. Gazzano, P. Samori, V. Palermo, *J Am Chem Soc* 2009, **131**, 15576-15577.

2. B. Lesiak, G. Trykowski, J. Toth, S. Biniak, L. Kover, N. Rangam, L. Stobinski, A. Malolepszy, *J Mater Sci* 2021, **56**, 3738-3754.

3. I. Iacoboni, F. Perrozzi, L. Macera, G. Taglieri, L. Ottaviano, G. Fioravanti, *J Biomed Mater Res A* 2019, **107**, 2026-2039.

4. V. Brusko, A. Khannanov, A. Rakhmatullin, A.M. Dimiev, *Carbon* 2024, **229**, 119507.

5. G. Fioravanti, A. Galante, P. Fattibene, L. Torrieri Di Tullio, S. Colacicchi, G. De Thomasis, F. Perrozzi, N. De Berardinis, G. Profeta, L. Ottaviano, M. Alecci, *Nanotechnology* 2024, **35**, 245101.

6. A. Kovtun, D. Jones, S. Dell'Elce, E. Treossi, A. Liscio, V. Palermo, *Carbon* 2019, **143**, 268-275.
